# Gastric Cancer (GC) with Peritoneal Metastases (PMs): An Overview of Italian PSM Oncoteam Evidence and Study Purposes

**DOI:** 10.3390/cancers15123137

**Published:** 2023-06-10

**Authors:** Paolo Sammartino, Giovanni De Manzoni, Luigi Marano, Daniele Marrelli, Daniele Biacchi, Antonio Sommariva, Stefano Scaringi, Orietta Federici, Marcello Guaglio, Marco Angrisani, Maurizio Cardi, Alessia Fassari, Francesco Casella, Luigina Graziosi, Franco Roviello

**Affiliations:** 1 CRS and HIPEC Unit, Pietro Valdoni, Umberto I Policlinico di Roma, 00161 Roma, Italy; 2 Upper GI Surgery Division, University of Verona, 37129 Verona, Italy; 3 Department of Medicine, Surgery, and Neurosciences, Unit of General Surgery and Surgical Oncology, University of Siena, 53100 Siena, Italy; 4 Advanced Surgical Oncology Unit, Surgical Oncology of the Esophagus and Digestive Tract, Veneto, Institute of Oncology IOV-IRCCS, 35128 Padova, Italy; 5 AOU Careggi, IBD Unit-Chirurgia dell’Apparato Digerente, 50100 Firenze, Italy; 6 Peritoneal Tumors Unit, IRCCS Regina Elena National Cancer Institute, 00144 Rome, Italy; 7 Peritoneal Surface Malignancies Unit, Fondazione Istituto Nazionale Tumori IRCCS Milano, 20133 Milano, Italy; 8 General and Emergency Surgery Department, Santa Maria Della Misericordia Hospital, University of Perugia, 06125 Perugia, Italy

**Keywords:** gastric cancer, peritoneal surface malignancies, cytoreductive surgery, peritoneal metastases, hyperthermic intraperitoneal chemotherapy, pressurized intraperitoneal aerosol chemotherapy

## Abstract

**Simple Summary:**

Peritoneal metastases (PMs), arising from gastric cancer (GC), are one of the most common patterns of synchronous and metachronous dissemination and are generally associated with a poor prognosis. New therapeutic modalities are being increasingly employed for such patients. Here, we provide an overview of the recent literature on this topic, along with two studies currently underway: one at Sapienza University of Rome and the other at the University of Verona, focusing on the use of neoadjuvant intraperitoneal chemotherapy in combination with a classical neoadjuvant systemic chemotherapy (SC). This overview emphasizes the results obtained using neoadjuvant intraperitoneal treatment, which may find a place not only in the Eastern world, where it now represents a standard of care, but also among Western practitioners.

**Abstract:**

Gastric cancer (GC) continues to be one of the leading types of malignancies worldwide, despite an ongoing decrease in incidence. It is the fifth most frequent type of cancer in the world and the fourth leading cause of cancer death. Peritoneal metastases (PMs) occur in 20–30% of cases during the natural history of the disease. Systemic chemotherapy (SC) is undoubtedly the standard of care for patients with GC and PMs. However, with the development of highly effective regimens (SC combined with intraperitoneal chemotherapy), significant tumor shrinkage has been observed in many patients with synchronous GC and PMs, allowing some to undergo curative resection “conversion surgery” with long-term survival. In recent years, there has been growing interest in intraperitoneal chemotherapy for PMs, because the reduced drug clearance associated with the peritoneal/plasma barrier allows for direct and prolonged drug exposure with less systemic toxicity. These procedures, along with other methods used for peritoneal surface malignancies (PSMs), can be used in GCs with PMs as neoadjuvant chemotherapy or adjuvant treatments after radical surgery or as palliative treatments delivered either laparoscopically or—more recently—as pressurized intraperitoneal aerosol chemotherapy. The great heterogeneity of patients with stage IV gastric cancer did not allow us to carry out a systemic review; therefore, we limited ourselves to providing readers with an overview to clarify the indications and outcomes of integrated treatments for GCs with PMs by analyzing reports from the international clinical literature and the specific experiences of our oncoteam.

## 1. Introduction

Gastric cancer (GC) is the fourth leading cause of cancer death and the fifth most frequently diagnosed cancer worldwide [1]. Peritoneal metastases (PMs) occur in 20 to 30% of cases during the natural history of the disease [2]. The results of the REGATTA trial showed that, in patients with stage IV GC with a single non-curable factor (in more than 70% of PMs), the initial removal of the primary tumor did not show any survival benefit compared with chemotherapy alone [3]. Therefore, the Japanese GC treatment guidelines recommend primary chemotherapy to improve the prognosis of patients with advanced GC and non-curative factors [4]. Oligometastatic and highly metastatic gastric cancer patients, both belonging to stage IV, have different treatment perspectives and prognoses. In the era of precision medicine and limited resources, the selection of candidates for an aggressive approach to stage IV gastric cancer has gained particular relevance. Patients with oligometastatic disease, represented by a single site and limited metastatic spread with diagnoses such as PAN+, <3 Liver M+, Cyt+, Krukenberg tumor, and peritoneal metastases with PCI < 6, showing a clinical response to intensive chemotherapy may benefit from aggressive multimodal treatment. In our opinion, this should not be a static definition, but rather a dynamic one that integrates a good response to chemotherapy and the possibility of achieving an R0 surgical resection.

Systemic chemotherapy (SC) is undoubtedly the standard of care for patients with GC and PMs in a palliative setting in Western countries as well [5]. However, the results are poor, the long-term control of PMs is rarely achieved—with median overall survival (OS) ranging from 8.0 to 13.2 months—and the objective response is difficult to evaluate [6]. The prognosis of patients with GC and synchronous PMs is especially poor as a result of their decreased response to SC, as well as the epidemiologic increase in pathological subtypes that are more frequently associated with the development of PMs [7] and more easily underestimated at the preoperative stage [8].

However, with the development of highly effective regimens (SC combined with intraperitoneal chemotherapy), remarkable tumor shrinkage has been observed in many patients with GC and synchronous PMs, allowing some patients to undergo a curative resection with long-term survival [9,10]. This strategy is referred to as “conversion surgery”, which is defined as a surgical treatment aimed at R0 resection after a good response to induction therapy for tumors originally considered unresectable for technical and/or oncological reasons [11].

In the last few years, there has been growing interest in intraperitoneal chemotherapy for PMs, as the reduced clearance of drugs due to the existence of the peritoneal–plasmic barrier allows for the direct and prolonged exposure of metastases with reduced systemic toxicity [12,13]. These procedures, together with other methods used in the treatment of peritoneal surface malignancies (PSMs), may be used in GC with PMs as neoadjuvant chemotherapy or adjuvant treatments after radical surgery (hyperthermic intraperitoneal chemotherapy, HIPEC) or as palliative treatments delivered either laparoscopically (LHIPEC) or—more recently—as pressurized intraperitoneal aerosol chemotherapy (PIPAC), which uses a specific laparoscopic device (Capnopen) to spread chemotherapy agents in the peritoneum [14]. The aim of this overview is to analyze the indications for and results of the integrated treatment of GC with PMs by examining clinical reports from the international literature and the specific experiences of our oncoteam.

## 2. Neoadjuvant Chemotherapy for Patients with GC and Synchronous PMs

A recent paper analyzed a series of selected patients with GC and synchronous PMs who underwent gastrectomy plus cytoreduction (CRS) and HIPEC, reaching a median OS of 11 years. Almost 80% of these 28 patients (6.2% of a broader case series) underwent neoadjuvant chemotherapy [15]. The likelihood of a curative surgical treatment thus seems to rely on the neoadjuvant treatment for the reduction or at least control of cancer spread, even though treatment schedules differ around the world. Western research groups seem to favor SC, while Eastern groups frequently use intraperitoneal chemotherapy. The use of neoadjuvant SC in patients with GC and synchronous PMs has recently been reported both by Western and Eastern groups, although the inclusion criteria, drugs, and clinical characteristics of the patients differ greatly. Two Japanese authors compared neoadjuvant SC followed by surgery to a primary surgical approach in locally advanced GC and reported similar overall and progression-free survival (PFS) [16,17]. However, although it considered patients with positive cytology or perigastric peritoneal involvement, the study by Yamaguchi [17] reported that a subgroup of patients with a complete peritoneal response after neoadjuvant SC showed a far better prognosis when compared with patients who underwent upfront surgery, and that the combination of Docetaxel, Cisplatin, and Fluoruracil was the most effective regimen. A European study [18] analyzed the effects of neoadjuvant SC (AIO-FLOT3) on a series of patients with GC (gastroesophageal junction included) and synchronous metastatic disease followed by surgery. Of the 187 patients included (127 with extensive and 60 with limited metastatic disease), 70 had PMs and only 2 of them underwent surgery.

In four papers from European authors about patients with GC and synchronous PMs who underwent gastrectomy plus CRS combined with HIPEC, most report the use of neoadjuvant SC, varying from 53% in Bonnot et al. [19] to 66% in Marano et al. [20] and 74% in Rau et al. [21] and reaching over 90% of cases in the paper of Manzanedo et al. [22].

In any case, the real role of neoadjuvant SC is questioned by some authors owing to non-homogeneous treatment protocols, uncertainty about the necessary number of treatment cycles (a higher than mean number seems to result in a worse outcome), and the lack of a significant prognostic outcome for the tumor regression score of the surgical specimen [23]. Nevertheless, an interesting point connected to the cited work from Yamaguchi [17] was reported by our oncoteam [20]; that is, not only did neoadjuvant SC carry a general positive prognostic effect, but no cancer cells were found in the peritoneal cytology at surgery in 85% of patients. These data underline that the role of neoadjuvant SC in patients with PMs from GC should be primarily directed to the control of the peritoneal disease to increase the rate of curative surgery and prevent local recurrence in most cases [24].

We generally refer to intraperitoneal chemotherapy as the HIPEC procedure, which is rarely utilized or proposed as a neoadjuvant treatment [25,26,27] and is most frequently—as with other PSMs—combined with CRS as an adjuvant treatment. However, based on the current results, we must consider that CRS combined with HIPEC is indicated in a limited number of patients with GC and PMs, particularly those with only minimal peritoneal involvement, although this has yet to be confirmed in larger trials [28]. The only way to increase the number of eligible patients and improve their prognosis thus seems to be the optimization of the neoadjuvant chemotherapy protocols. For this reason, Western authors have also shown increasing interest [29,30] in neoadjuvant normothermic intraperitoneal treatments, with or without SC, which have been used for many years in Eastern medicine.

Yutaka Yonemura was the first to propose a neoadjuvant treatment including systemic and intraperitoneal chemotherapy associated with oral Fluoruracil for patients with GC and PMs to reach a downstaging of the peritoneal disease with negative peritoneal cytology, aiming at a subsequent gastrectomy with CRS combined with HIPEC [31]. Since its initial introduction, neoadjuvant intraperitoneal and systemic chemotherapy (NIPS) has become the current conversion therapy for GC with PMs in Eastern countries, particularly China and Japan, with protocols based almost entirely on intraperitoneal paclitaxel because of its specific favorable pharmacokinetics [32]. In the last few years, several phase II trials have been published—mainly by Japanese authors—using intraperitoneal paclitaxel or docetaxel combined with oral Fluoruracil and systemic cisplatin, oxaliplatin, or paclitaxel, reaching a 1-year survival rate close to 80% and a median survival of 18–25 months. At the end of the NIPS treatment, the intraperitoneal cytology was negative in 85% of the patients and ascites decreased in over 60% of the patients, with a conversion surgery rate of between 40 and 60% of patients (Table 1).

The key point of an indication for surgery is in any case the achievement of negative peritoneal cytology, as supported by Western authors [44]. Of particular interest is a recent paper from Yonemura et al. [42]. In a series of 419 patients treated for GC and PMs from 2006 to 2019, the authors showed that NIPS allowed complete CRS (CC0) in 63.5% of cases, with a median survival of 20.5 months and a 10-year survival of 8.3%.

Thus far, only one randomized study has been published on this topic (the PHOENIX-GC Trial) [45], which failed to demonstrate a significant increase in survival between patients with GC and PMs undergoing intraperitoneal and intravenous Paclitaxel plus S-1 and those given intravenous Cisplatin plus S-1. These results are likely due to the critical imbalance between the groups, as fewer patients with limited PMs and more patients with advanced PMs were enrolled in the NIPS arm. Further data analysis adjusted for baseline ascites, and excluding patients with post-protocol violations showed a significant increase in the 3-year survival rate (21.9% vs. 6%) in the group of patients who underwent NIPS. Despite the fact that surgery was not mandatory after neoadjuvant treatment in the trial and was only recommended in the case of a good response, 46% of the patients in the NIPS group and 22% in the SC group underwent surgery after responding to neoadjuvant treatment, with a survival of 32 and 25 months, respectively. Although the influence of surgery on survival is difficult to evaluate, it can be affirmed that a higher number of patients could benefit from the surgical treatment in the NIPS group when compared with the SC neoadjuvant group [46].

A recent paper from China used a propensity-score-matched analysis to compare patients with peritoneal metastases from gastric cancers (including positive peritoneal cytology without detectable lesions within the peritoneal cavity) that underwent NIPS (intraperitoneal docetaxel plus intravenous docetaxel + oxaliplatin and oral S1) to patients receiving SC (intravenous docetaxel + oxaliplatin and oral S1) [47]. Despite some limitations, i.e., a single-center retrospective study with a small sample, NIPS yielded better overall survival than SC. A subgroup analysis indicated that NIPS showed a survival benefit in patients with limited peritoneal disease whose cytology turned negative and who underwent conversion surgery.

A Chinese group [48] recently published the protocol of a new randomized trial that will compare NIPS (intraperitoneal and systemic Paclitaxel plus oral Fluoruracil) and SC in combination with Paclitaxel and oral Fluoruracil in patients with synchronous PMs from GC after a laparoscopic evaluation (DRAGON 01 Study ChiCTR-IIR-16009802). This study seems to have a better design than the PHOENIX-GC study owing to the homogeneity of the drugs used and the endpoints considered (pathological response and the rate of conversion surgery). However, the inclusion criteria are unclear given that they do not take into consideration a cutoff in the PCI, the extent of ascites, or HER2 status, which is considered by many authors as an exclusion criterion for NIPS treatment [30,49,50].

## 3. CRS Combined with HIPEC in Patients with GC and PMs

In the field of PSM, the most controversial aspects of integrated treatments using CRS and HIPEC regard the management of patients with PMs from GC. To date, only one randomized study—with a limited number of patients—has been completed comparing CRS combined with HIPEC to CRS alone in patients with clinically evident PMs [51]. The study showed better survival in the HIPEC group (11 vs. 6.5 months, *p* = 0.04), which was more significant in the subgroup of patients with synchronous PMs (*p* = 0.02). A multivariate analysis showed that use of HIPEC, synchronous metastases, the completeness of the cytoreduction score, and neoadjuvant chemotherapy were significantly associated with a better prognosis. Surprisingly, the peritoneal cancer index (PCI) was not statistically significant.

Several non-randomized studies, mostly multicentric, studying the impact of CRS combined with HIPEC in the treatment of patients with PMs from GC have been published in recent years [19,20,21,22,42,52,53]. Most of these papers confirm the prognostic importance of the already cited parameters, but also show the importance of the PCI in selecting patients suitable for surgery, which would be reserved for those with a PCI ≤ 7. Patients with a higher PCI, particularly those with specific histotypes (poorly cohesive), are not considered ideal candidates for CRS and show a poor outcome.

Currently, because of the lack of large RCTs in the Western world, there are no guidelines recommending the use of CRS combined with HIPEC in the treatment of PMs from GC. Despite the fact that the Peritoneal Surface Oncology Group International supports it (see http://www.psogi.com/psogi/international-recommendations-for-the-management-of-peritoneal-metastases/, accessed on 1 January 2021), further studies are needed.

Similar to other sections of PSM treatment, the crucial issue is the role of HIPEC in the integrated management of PMs from GC or its use for the prevention of metachronous peritoneal involvement. The role of HIPEC combined with CRS versus CRS alone was investigated in a recently published meta-analysis [54]. In this meta-analysis, despite the small number of studies included, the treatment of patients with PMs from GC with CRS combined with HIPEC showed higher overall survival and a lower percentage of peritoneal recurrence compared with CRS alone, with similar complication rates in both groups. The use of CRS combined with HIPEC in the treatment of clinically evident PMs, or the possible benefits of HIPEC alone combined with surgery for locally advanced GC, possibly with only simple positive cytology in the peritoneum, were studied in another meta-analysis by Granieri S et al. [55]. As one would expect, the favorable impact of HIPEC was more evident when associated with the surgical treatment of locally advanced GC when compared with patients in whom it was used in combination with CRS for the treatment of clinically evident PMs. In this latter group of patients treated with curative intent, the completeness of cytoreduction represents a crucial step, where HIPEC combined with CRS is an independent predictor of better prognosis, with a 2.6-fold increase in survival outcome.

The benefit of prophylactic HIPEC in advanced gastric cancer is indicated by the high incidence of metachronous peritoneal metastases in 30 to 60% of patients, depending on serosal involvement and specific histotypes (signet ring cells) [56,57,58]. For these patients with metachronous peritoneal spread, a treatment with a curative aim is difficult to propose. Many studies have suggested that HIPEC be used as an adjuvant treatment after surgery in GC patients at high risk for PMs. In these patients, HIPEC performed after radical surgery with no peritoneal disease has shown good results [59,60,61]. Randomized studies on this topic have been proposed; however, their results have not been published yet. In 2014, Glehen et al. [62] proposed a randomized study of locally advanced GC (T3/T4 with eventual positive cytology), comparing gastrectomy and D2 lymphadenectomy, with or without HIPEC with oxaliplatin, stratified according to the treatment center and pathology (GASTRICHIP trial NCT01882933). A similar trial (HIPEC- 01 NCT0235676) is ongoing in China; in both, the primary outcome is 5-year survival. Another trial has been proposed by German authors [63]. After neoadjuvant SC with FLOT, this study will randomize diffuse or mixed type gastric cancers, including Siewert type II and III tumors without peritoneal disease, into two arms: Surgery + Adjuvant FLOT vs. Surgery + HIPEC + Adjuvant FLOT (NCT04447352).

Many European groups have proposed randomized studies analyzing the role of CRS combined with HIPEC in GC with clinically evident PMs. The German trial GASTRIPEC (NCT02158988) randomized patients with GC and synchronous PMs who underwent pre- and postoperative SC into two groups, stratified for PCI and HER2 status. In the first group, patients underwent CRS with curative intent; in the second, patients received CRS plus HIPEC (Mytomicin C 15 mg/m^2^ + Cisplatin 75 mg/m^2^ per 60 min). The results did not show any difference in OS (median OS: 14.9 months in both arms), although a subgroup analysis showed a significant increase in survival in the HIPEC group when a complete CRS had been achieved. In addition, PFS was significantly longer in the HIPEC arm than with CRS alone (7.1 vs. 3.5 months *p* = 0.04). The trial was stopped because of the slow rate of patient recruitment, and only 105 of the 180 planned cases were included. It is interesting to note that 55 patients could not reach surgical treatment owing to disease progression or because they died of disease, questioning the limits of neoadjuvant SC in these patients [64].

Another interesting and well-structured study is the Dutch study PERISCOPE from the Netherlands Cancer Institute, carried out in two phases. PERISCOPE I [65] included a heterogenous group of patients with locally advanced GC (cT3/cT4) with peritoneal positive cytology as well as GC patients with PMs limited to the sovramesocolic area or with a single pelvic implant, with adequate performance status and after neoadjuvant SC. Among the exclusion criteria were metachronous PMs. The primary goal of the study was to investigate the role of gastrectomy combined with CRS and HIPEC after neoadjuvant SC. The secondary goal was to assess the maximal tolerated dose of docetaxel to be given in normothermia (90 min) at the end of 30 min of HIPEC with 460 mg/m^2^ oxaliplatin. The results were reported in two consecutive papers [66,67] for an extremely limited number of cases, as 12 of the 37 patients included showed disease progression and could not undergo resection. The authors defined 50 mg/m^2^ as the maximal tolerated dose of docetaxel and, at a median follow-up of 37 months, reported a median OS and PFS of 15 months (0–53) and 12 months (0–29), respectively. The focal points of the trial were the minimal or no response to neoadjuvant SC, histologically proven in 40% of the patients, and the significant rate of microscopic rather than radical resection (R1) at pathology in 28% of the patients. We agree with the authors that the high percentage of the diffuse type of cancer (68%) may be an explanation for such poor results; however, at the same time, it should be emphasized that this particular histotype, currently better defined as poorly cohesive carcinoma (PCC) [68], requires specific attention because of its increasing incidence in Western countries [69], its specific tendency for peritoneal spread, and the characteristics of its submucosal growth pattern that frequently result in the preoperative underestimation [70] of its spread.

Based on the results of PERISCOPE I, the Dutch authors developed the PERISCOPE II trial (NCT03348150) to investigate if neoadjuvant SC followed by gastrectomy and CRS with HIPEC could have a survival advantage in selected patients with GC and PMs when compared with palliative SC, which, at the moment, is the standard of treatment in the Netherlands [71]. In this trial, a laparoscopy is performed before inclusion in the study to assess the resectability of the primary cancer (cT3-4) and/or limited peritoneal disease or a simple positive cytology at peritoneal lavage. After two to three cycles of neoadjuvant SC, freely decided by each participating center, the patient’s response is evaluated using a CT scan. The responders are then included in the study and patients are stratified according to treatment center, histotype (intestinal or diffuse), and the extent of peritoneal spread (macroscopic PMs vs. positive peritoneal cytology). For patients allocated to the experimental arm, a new preoperative CT scan is performed, followed by explorative laparotomy in patients with stable disease. If the PCI is <7, a gastrectomy with D2 lymphadenectomy and a CRS aiming to remove all visible peritoneal disease followed by HIPEC is performed.

Although the study has a good basic rationale, particularly regarding the aim to assess the advantage of an integrated treatment over palliative SC in selected cases, in our opinion, the trial has important limitations. The first is that a CT scan does not seem to be the best diagnostic tool to evaluate the extent of the peritoneal disease within such a rigid PCI cutoff (PCI below or over 7) [72]. A laparoscopic evaluation would likely be more accurate, avoiding useless laparotomies than can negatively impact further treatments. In addition, it is important to assess the persistence of a positive cytology, a criterion that could itself contraindicate a surgical approach, despite the use of HIPEC.

## 4. Pressurized Intraperitoneal Aerosol Chemotherapy (PIPAC)

PIPAC is a novel mode of intraperitoneal drug delivery for patients with PMs of various origin that reached widespread use with the standardization of the procedure and ongoing specific training courses [73]. A recent review by Alyami et al. [74] reported on a significant number of procedures (1800 on 800 patients) and concluded that PIPAC is safe, giving encouraging therapeutic results with a good quality of life. Further phase I, II, and III trials are still needed to assess exact indications. However, in one of the largest series of patients with PMs from GC who underwent PIPAC [75], a large bias was reported based on the clinical indications for which the procedure was adopted, which makes it difficult to evaluate clinical outcomes. The majority of the patients underwent multiple treatments for metachronous peritoneal metastases and intractable ascites, an indication for which PIPAC seems to have little impact. Moreover, the evaluation of the PCI remains inaccurate and repeated peritoneal biopsies to assess the peritoneal regression grading score (PRGS) do not seem to correlate with the prognosis. The only prognostic factor is the number of treatments (≥3); however, this factor has a bias related to the fact that the clinical conditions of the patients with more advanced disease prevent multiple treatments. Furthermore, only 7% of the patients underwent gastrectomy with CRS and HIPEC.

Better clinical results would be reached with the early use of PIPAC and an improved patient selection process, as reported in another study from Alyami et al. [76]. This paper showed that 21.6% of patients with PMs from GC, previously considered unresectable, underwent CRS combined with HIPEC after three cycles of PIPAC. The paper also reported the results of a group of patients with various primary tumors; however, most of them underwent a single chemotherapy line, had no ascites, and had a low PCI. These results seem to suggest a role of PIPAC not only in palliation, but also in selected patients as a neoadjuvant treatment.

## 5. Metastatic GC (MGC): An Overview

In Western countries (USA and Europe), synchronous metastatic disease is diagnosed in 30% of patients, with reported survival varying from a few months to one year depending on the site of the metastases and the treatments; at the moment, there are no guidelines for the different clinical pictures [18,77,78,79,80].

Although the existing data also show poor results for palliative surgery [79], SC combined with a surgical treatment that includes not only gastrectomy but also treatment for the metastatic disease seems to provide promising results according to Western and Eastern studies [78,81]. These last two papers are of particular interest because they were published in the same period and they report the results of a significant number of patients, although retrospectively evaluated, and the results tend to justify broader indications for integrated treatments in patients with MGC. Berger et al. [78] analyzed data from the National Cancer Database (NCDB) from 2004 to 2016 (1500 hospitals included) and considered three main sites of distant metastases (extra regional lymph nodes, liver, and peritoneum), showing a clear survival advantage for a complete CRS including gastrectomy and metastatic disease removal. Although significant, these results reflect the limits of the NCBD, which does not report the number and size of the metastatic disease. Moreover, more than 80% of the patients were considered M0 at preoperative staging, presuming an initial metastatic disease, while 55% of the patients underwent only postoperative chemotherapy.

Yoshida et al. [81] reported on a smaller number of cases after gathering information on conversion surgery experiences in 55 Asian centers (Japan, Korea, and China), supplying more information on a homogeneous patient sample based on a new pathologic classification of MGC [82], while also taking into consideration only MGC patients operated upon after SC. The first significant result is the acceptable rate of major surgical complications (20.6% Grade II or higher according to Clavien–Dindo). Moreover, the study reports a high median survival in patients who underwent R0 conversion surgery in all of the considered categories, not only in patients initially deemed to have resectable metastatic disease, suggesting a different surgical approach for all patients with a stage IV GC. Induction chemotherapy could thus be considered in every case of stage IV GC, acting as an NACT in resectable patients and as palliative care in others, monitoring the treatment response for a possible R0 surgical treatment in any case. Another main result is the clinical application of a so-called biological stage IV GC classification, differentiating ab initio the patients with a macroscopic peritoneal involvement, which carries a worse outcome. Those patients, also including the patients with a positive peritoneal cytology, accounted for 46.6% of the entire series.

These data, however, come from a selected group of surgical patients and thus may not represent the complete picture. In the last two AJCC Classifications (VII and VIII edition), patients with a positive peritoneal cytology are considered as stage IV; in addition, although the laparoscopic collection of peritoneal fluid is recommended in Western guidelines for all potentially resectable patients with stage > cT1b (NCCN Clinical practice guidelines in oncology Gastric Cancer Version 2.2022 [5]), it is not clear how many patients actually undergo this procedure. The real benefit of a diagnostic laparoscopy in clinically M0 patients with GC before radical surgery is still a matter of debate, in light of both recent Eastern guideline indications [83,84] and the different methods used for the detection of peritoneal cancer cells [85,86].

Although different surgical groups have proposed selective criteria for the use of diagnostic laparoscopy, a positive peritoneal cytology even without macroscopic peritoneal disease seems to carry a poor prognosis in both Western and Eastern experiences, highlighting the positive role of chemotherapy in these patients [87,88]. An English meta-analysis provides important information on this matter, reporting a better prognosis in GC patients who are clinically M0 but who have a positive peritoneal cytology that becomes negative after neoadjuvant chemotherapy. However, in 25% of the patients with a negative peritoneal cytology, this finding may turn positive during NACT delivered for other metastatic sites. Hence, a diagnostic laparoscopy is needed not only at initial staging, but also at restaging [89].

## 6. Experience and Proposals of the Italian PSM Oncoteam

Marano et al. [20] recently published the experiences of the Italian PSM oncoteam with an integrated treatment involving CRS combined with HIPEC in 90 patients with GC and synchronous PMs. The adoption of a neoadjuvant SC and the absence of cancer cells in the peritoneal cytology at operation were critical for a better outcome. Given these results, we tried to understand how, through the optimization of neoadjuvant treatments, to achieve (a) an extension of the selection criteria to include these patients for a curative treatment, (b) an increase in the rate of patients eligible for surgery, and finally (c) an improvement in outcome. Two studies are currently ongoing, one at Sapienza University of Rome and the other at the University of Verona; both are focused on the use of intraperitoneal neoadjuvant chemotherapy combined with a classic neoadjuvant SC. Both studies include patients with GC and synchronous PMs not previously treated and the use of PIPAC, whereas the inclusion criteria and treatment plans are different.

## 7. Sapienza Phase II NIPS Study (Grant Sapienza n RG12117A807F5D85)

This study is very similar to the bidirectional treatment first proposed by Yonemura et al. [31] with some differences, including the use of capecitabina per os instead of the S-1 used in the Japanese study; induction PIPAC with cisplatin and doxorubicin at the staging laparoscopy; and, above all, the use of the same protocol as adjuvant treatment in patients submitted to CRS plus HIPEC, in order to prevent peritoneal recurrence as much as possible. Patients considered for the study are those with GC according to the last AJCC Classification (including Siewert III tumors) with synchronous macroscopic PMs (excluding patients with only positive cytology), with the absence of her2/neu overexpression, and without extraperitoneal metastases.

**Figure 1:**
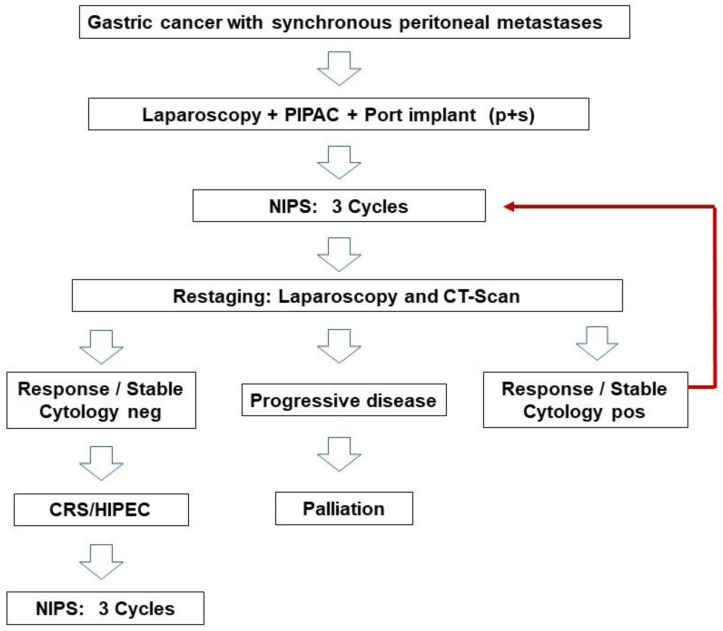
Design of the study.

As shown in Figure 1, according to the design of the study, patients undergo a first staging laparoscopy combined with PIPAC and ports’ implant after diagnosis. After three cycles of NIPS, patients undergo a CT scan and restaging laparoscopy, after which those considered responders or with stable disease and negative peritoneal cytology become candidates for surgery. Patients considered responders but with positive peritoneal cytology are submitted to further NIPS cycles. Patients with progression of the disease leave the study.

The inclusion criteria are age between 18 and 70 years; ECOG PS 0–1; PCI < 15 at staging laparoscopy; patients not previously treated for the current disease; patients with adequate bone marrow, liver, and renal function; patients able to have an adequate caloric intake and with life expectancy >3 months; and patients able to give informed written consent. The exclusion criteria are ascites > 500 mL; distant metastases (liver, lung, brain, or bone) or paraaortic and/or extraregional lymphatic spread; other cancer diagnoses in the last 5 years (excluding cutaneous basalioma or preinvasive cervical carcinoma); and pregnancy or breastfeeding status or other systemic illness preventing inclusion in the protocol.

## 8. PIPAC VEROne—A Randomized Multicenter Phase III Trial: Trial Registration EUDRACT 2021-000830-33; NCT 05303714

This PIPAC VEROne (pressurized intraperitoneal aerosol chemotherapy in multimodal therapy for patients with oligometastatic peritoneal GC) trial, recently published by some members of our oncoteam [90], is part of the study of a neoadjuvant treatment modality that can increase the number of patients who, although suffering from peritoneal metastases, can benefit from curative surgical treatment. This study, similar to the previous one, combines systemic neoadjuvant treatment with endoperitoneal neoadjuvant treatment in the experimental arm and compares it to a control arm of systemic-only neoadjuvant treatment with FOLFOX as a phase III study.

However, there are major differences between the two studies in terms of both inclusion criteria and the methodology of the administration of intraperitoneal neoadjuvant chemotherapy.

The first difference regarding the inclusion criteria is the inclusion of cases with simple positive cytology alongside patients with macroscopically evident peritoneal spread, although limited to a PCI < 6. With the first staging laparoscopy and the following three laparoscopic accesses for the execution of PIPAC, which intersperses the systemic chemotherapy, one arrives, with the restaging laparoscopy required for the final evaluation, at a total of five laparoscopic accesses. This may lead to a possible localization of the disease in the abdominal wall for these patients, especially if they are affected by diffuse histotypes (poorly cohesive subtypes) with a marked peritoneal tropism. Then, with regard to the final laparoscopic restaging (foreseen only in the experimental arm), it appears rather unusual how, before proceeding with a possible surgical treatment, the need for the verification of endoperitoneal cytological negativity is not specified. Of particular interest in the study is the histopathological evaluation of the response to treatment that can add to the meaning of an intraoperative PCI, which, especially after neoadjuvant chemotherapy, may not be particularly reliable. Also of importance is the assessment of the patients’ quality of life associated with a cost–benefit analysis of the procedure.

## 9. Conclusions

**Figure 2:**
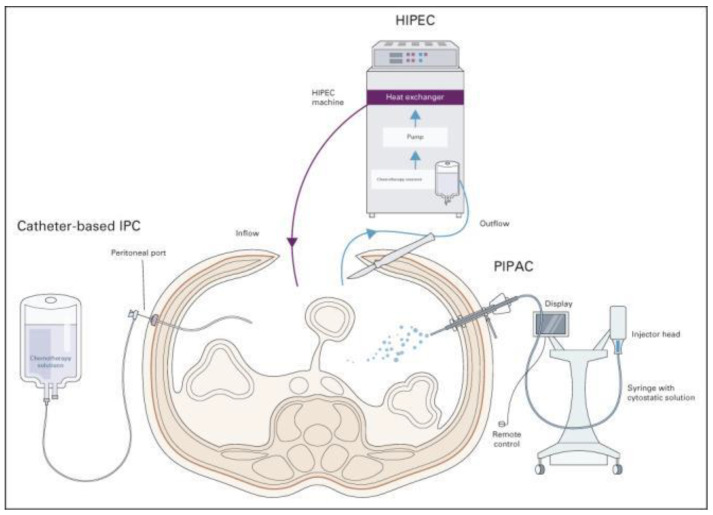
Peritoneal-directed modalities and their roles in treatment and prophylactic strategies for GCPM. Catheter-based IPC has been evaluated in both adjuvant and neoadjuvant settings in the management of GCPM. The adjuvant combination of systemic and intraperitoneal chemotherapy may help downstage PM, allowing for conversion gastrectomy, whereas the role of adjuvant early postoperative intraperitoneal chemotherapy in the prevention of metachronous PM remains unclear. HIPEC is most commonly carried out in conjunction with cytoreductive surgery as a potentially curative strategy in patients with low-volume PM and potential for complete cytoreduction. A potential role exists for prophylactic HIPEC in patients with GC undergoing gastrectomy to prevent or reduce metachronous PM recurrence, with ongoing studies currently underway. Studies on PIPAC have thus far been limited to palliative treatment for patients with PM; the role of PIPAC in the treatment of GCPM requires further evaluation and is currently limited to the settings of clinical trials. GCPM—gastric cancer peritoneal metastasis; HIPEC—hyperthermic intraperitoneal chemotherapy; IPC—intraperitoneal chemotherapy; PIPAC—pressurized intraperitoneal aerosol chemotherapy.

The peritoneum is a common target of metastatic disease in advanced GC. Despite an earlier diagnosis commonly obtained by diagnostic laparoscopy, its aggressiveness and resistance to traditional SC, which remains the current standard of care according to most international guidelines, leads these patients to a dismal prognosis. An improvement in outcome could be achieved through advances in the genomic and molecular profiling of the disease combined with an integrated therapeutic strategy of locoregional and SC. New modalities of intraperitoneal chemotherapy, such as open or closed hyperthermic intraperitoneal chemotherapy (HIPEC), intraperitoneal chemotherapy (IPC), and pressurized intraperitoneal chemotherapy with aerosol (PIPAC), are currently being used in various countries, and it is likely that further studies will consider the incorporation of peritoneal-directed treatment with systemic therapy (Figure 2) [91].

## Figures and Tables

**Table 1:** Clinical outcomes of repeated IPC with systemic chemotherapy for gastric cancer with PM.

Author, Year	IP Regimen	Systemic Regimen	Study	n	MST (mo)	1 y OS (%)	Cytology Negative Conversion Rate (%)
Ishigami, 2010 [33]	PTX (20 mg/m^2^)	S-1 + PTX	P2	40	22.5	78	86
Fujiwara, 2012 [34]	DTX (40~60 mg/m^2^)	S-1	R/S	18	24.6	76	78
Fushida, 2013 [35]	DTX (45 mg/m^2^)	S-1	P1/2	39	16.2	70.4	81
Yamaguchi, 2013 [36]	PTX (20 mg/m^2^)	S-1 + PTX	P1	35	17.6	77.1	97
Ishigami, 2016 [37]	PTX (20 mg/m^2^)	S-1 + PTX	P3	114	17.7	71.9	95
Fujiwara, 2016 [38]	PTX (40 mg/m^2^)	S-1 + L-OHP	P2	60	NR	71.5	71
Fukushima, 2017 [39]	DTX (10 mg/m^2^)	Cap + CDDP	P2	48	NR	75	76
Cho, 2017 [40]	DTX (100 mg/m^2^)	Cap + CDDP	P1/2	39	15.1	-	-
Shinkai, 2018 [41]	PTX (60 mg/m^2^)	S-1 + PTX + CDDP	P2	17	23.9	82.4	-
Yonemura, 2020 [42]	DTX (30 mg/m^2^) + CDDP (30 mg/m^2^)	DTX + CDDP	R/S	419	20.5	70	63.5
Saito, 2021 [43]	PTX (40 mg/m^2^)	S-1 + L-OHP	R/S	44	25.8	79.5	86

Cap—capecitabine; CDDP—cisplatin; DTX—docetaxel; IPC—intraperitoneal chemotherapy; L-OHP—oxaliplatin; MST—median survival time; NR—not reached; OS—overall survival; PM—peritoneal metastases; PTX—paclitaxel; P1, 2, 3—phase I, II, III; R/S—retrospective study; -—not described.

## Data Availability

The data presented in this study are available on request from the corresponding author. The data are not publicly available due to privacy reasons related to the neoplastic pathology under study.
